# CLASS: constrained transcript assembly of RNA-seq reads

**DOI:** 10.1186/1471-2105-14-S5-S14

**Published:** 2013-04-10

**Authors:** Li Song, Liliana Florea

**Affiliations:** 1 Department of Computer Science, Johns Hopkins University, Baltimore, MA 21218, USA; 2 McKusick-Nathans Institute of Genetic Medicine, Johns Hopkins University School of Medicine, Baltimore, MA 21205, USA

## Abstract

**Background:**

RNA-seq has revolutionized our ability to survey the cellular transcriptome in great detail. However, while several approaches have been developed, the problem of assembling the short reads into full-length transcripts remains challenging.

**Results:**

We developed a novel algorithm and software tool, CLASS (Constraint-based Local Assembly and Selection of Splice variants), for accurately assembling splice variants using local read coverage patterns of RNA-seq reads, contiguity constraints from read pairs and spliced reads, and optionally information about gene structure extracted from cDNA sequence databases. The algorithmic underpinnings of CLASS are: i) a linear program to infer exons, ii) a compact splice graph representation of a gene and its splice variants, and iii) a transcript selection scheme that takes into account contiguity constraints and, where available, knowledge about gene structure.

**Conclusion:**

In comparisons against leading transcript assembly programs, CLASS is more accurate on both simulated and real reads and produces results that are easier to interpret when applied to large scale real data, and therefore is a promising analysis tool for next generation sequencing data.

**Availability:**

CLASS is available from http://sourceforge.net/projects/splicebox.

## Introduction

Gene annotation is the first and most important step in analyzing a genome. More than 90% of human genes [1,2] are alternatively spliced to produce multiple mRNA transcripts involving different combinations of exons. The number of splice variants of a gene varies, from two to possibly thousands [3]. Recently, the RNA-seq technology has made it possible to survey the cellular transcriptome at unprecedented depth, within days and at a fraction of the cost of traditional methods. However, assembling the short reads into full-length transcripts is a difficult problem, complicated by artifacts in sample preparation, sequencing and read alignment.

Programs that assemble transcripts from short RNA-seq reads aligned to a reference genome largely follow two approaches [4]. In the *first *approach, programs such as Cufflinks [5] and Scripture [6] use read alignments to predict the exon-intron structure of transcripts, then employ statistical models of fragment distributions to quantify their expression levels. To predict transcript models, Cufflinks represents all fragments at a locus as an overlap graph in which two reads are connected if they overlap and have compatible splice patterns, and then traverses the graph to produce the minimum number of transcripts that can explain all the input fragments. This minimization approach may result in under-prediction. In contrast, Scripture enumerates combinations of exons from spliced reads within windows of the gene, and then assembles them into whole transcripts. Thus, Scripture may produce many more isoforms than present in the sample. In the *second *approach, programs such as IsoLasso [7] and its recent implementation IsoCEM perform simultaneous assembly and quantification of transcripts, jointly modeling the two problems into a quadratic or an estimation maximization program.

There are several drawbacks to these approaches. First, programs that simultaneously predict transcript structure and estimate their abundance typically make unrealistic assumptions about the uniformity of read coverage along the length of the gene, which can lead to incorrect transcript models. Second, by reconstructing transcripts from RNA-seq data alone, programs overlook existing knowledge about the gene structure that can be used to more accurately infer isoforms [8].

We describe a novel algorithm, called CLASS (Constraint-based Local Assembly and Selection of Splice variants), for transcript reconstruction from RNA-seq data, which takes advantage of local read coverage patterns and known transcript substructures from existing annotations. CLASS employs a linear program to locally reconstruct combinations of exons represented in the RNA-seq data, then connects the exons into a splice graph [9]. A *splice graph *is a directed acyclic graph in which exons represent the *nodes*, introns derived from splice read alignments represent *edges*, and candidate transcripts are maximal paths in the graph [10]. CLASS then selects a subset of transcripts that parsimoniously explain all contiguity constraints derived from mate pairs and optionally incorporates gene structure knowledge from existing databases. This step is modeled as a SET_COVER problem. CLASS does not estimate transcript abundance; rather, once a set of transcripts is produced, any of a number of programs (e.g., cuffdiff2 [11] and RSEM [12]) can be used to rigorously quantify them.

We tested CLASS and three other popular programs, Cufflinks, Scripture and IsoCEM, an expectation-maximization variant of IsoLasso. CLASS was both more sensitive and more precise on simulated data. On a large real data set, namely the adrenal sample from Illumina's Human Body Map Project, CLASS had higher accuracy as measured by the F-value and produced results that were easier to interpret.

CLASS also has several ancillary advantages over current approaches, in particular single-stage transcript prediction and quantification methods such as those implemented in IsoLasso and SLIDE [8]. When linear programming is applied to a portion of the gene, to find exons rather than full transcripts, it leads to smaller systems that can be solved more easily and more accurately. Lastly, by decoupling the exon prediction, transcript selection and transcript quantification stages, CLASS allows for a modular design where each step can be performed by a variety of methods, including approaches developed elsewere.

The rest of the material is organized as follows. Section 2 introduces the linear program model for predicting exons from RNA-seq data. Section 3 describes the splice graph construction, and the subsequent enumeration and selection of candidate transcripts. Lastly, section 4 presents the results of evaluating CLASS and other programs on simulated and real data.

## Constraint-based local assembly of exons

**Figure 1:**
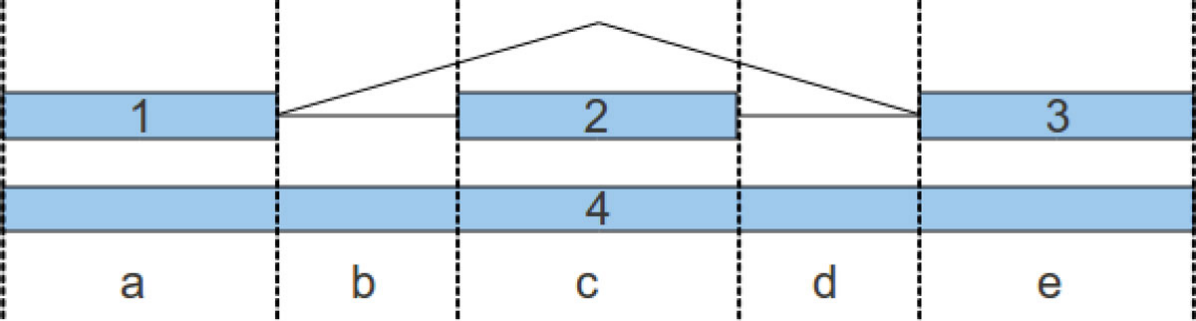
**Region, intervals, exons and subexons**.

We assume read coverage levels to be relatively uniform locally, and use this property to infer combinations of exons. We start by determining *regions *of the genome that are covered by reads and therefore represent exons or combinations of exons. We use the splice sites in each region to split the region into non-overlapping *intervals*, each belonging to one or possibly several exons. For instance, the region in Figure 1 has six possible exons (only four are shown, 1, 2, 3, 4) and can be split into five intervals *a, b, c, d *and *e*. Each interval can belong to more than one exon. We will refer to the portion of an exon corresponding to an interval as a *subexon*, for example 1, *a *or 4, *c*. We enumerate all possible combinations (subsets) of exons by pairing 5' splice sites with 3' splice sites ends, separately for the forward and the reverse strand, and score each combination using a linear optimization program. The best scoring combination of exons is chosen in the end.

Each feasible combination of candidate exons must satisfy the constraints:

1. Each 5' and 3' splice site must appear in at least one candidate exon.

2. Every read must be compatible with the exons in the current combination. For unspliced reads, the read must be included in some exon, whereas spliced reads must have splice junctions compatible with the group of exons.

3. For paired-end reads, the inner endpoints of the reads in a pair must either belong to the same exon, or must be connected by a path of non-overlapping exons from the current subset (*i.e*., the two mates must have compatible alignments).

For each combination that satisfies the conditions, we build a linear programming system. Denote *c_i,j _*the *average coverage *of exon *i *on interval *j*, defined as the average number of reads per base of subexon *i, j*. Let ${\bar{C}}_{j}$ be the average coverage on interval *j*. We define four types of constraints, and suppose we are processing the candidate exon subset 1, 2, 3, 4:

1. *Additivity: *The average coverage in each interval should be roughly equal to the sum of average coverage values for all subexons within that interval, e.g.:

$$|c_{1,a}+c_{4,a}-{\bar{C}}_{a}|\leq \varepsilon_{a}$$

2. *Continuity: *The average coverage of adjacent subexons of the same exon should be approximately equal, e.g.:

$$|c_{4,a}-c_{4,b}|\leq \varepsilon_{4}$$

3. *Conservation: *The total coverage of all subexons should be approximately equal to the total coverage of the region:

$$\overset{4}{\underset{i=1}{\sum }}\overset{e}{\underset{j=a}{\sum }}c_{i,j}l_{i,j}=\overset{e}{\underset{j=a}{\sum }}{\bar{C}}_{j}L_{j},$$

where *l_i,j _*is the length of subexon *i, j *and *L_j _*is the length of interval *j *(in practice, *l_i,j _*= *L_j_*).

4. *Non-negativity: *The average read coverage for each subexon should be no less than 1, e.g.:

$$c_{1,a}\geq 1$$

The objective function is to minimize $\sum \varepsilon_{n}$. Using this value, we calculate a final score for the exon combination. For single-end read data, the objective value is also the final score, whereas for paired-end data we add a penalty that takes into account whether the current exon subset satisfies the fragment length constraint:

5. *Read pair feasibility: *Suppose there are *N *read pairs, and *n_i,j _*read pairs where the left read starts in interval *i *and the right read ends in interval *j*. Assuming the average length of a fragment is *f_l _*and the standard deviation is *f_σ_*, we compute the minimal and maximal distance between interval *i *and interval *j *given the current exon subset. If the range of feasible distances is outside of the interval *f_l _*± 2*f_σ_, *then we add to the score $\frac{n_{i,j}}{N}$, or the proportion of unsatisfied pairs.

We solve the problem for *c_i,j _*and *ε_n_*. Quantities ${\bar{C}}_{j}$and *L_j _*are known or can be calculated from the input alignments. Finally, we choose the combination with the minimal score as the set of exons for the region. If there are multiple such combinations, we select the one with the smallest number of exons. Note that the *additivity *condition is similar to those used by IsoLasso and SLIDE, albeit formulated in terms of exons rather than transcripts, whereas the rest of the conditions are specific to our method.

## Candidate transcript enumeration and selection

Once the set of exons is identified, we generate a splice graph by connecting the exons (nodes) via introns (edges) extracted from spliced read alignments [9]. Candidate transcripts are encoded in the graph as maximal paths from a node with no incoming edges (*source*) to a node with no outgoing edges (*sink*). However, not all variants encoded in the graph will be real, and therefore we use the following SET_COVER formulation to select a high-confidence set of candidate transcripts.

**Figure 2:**
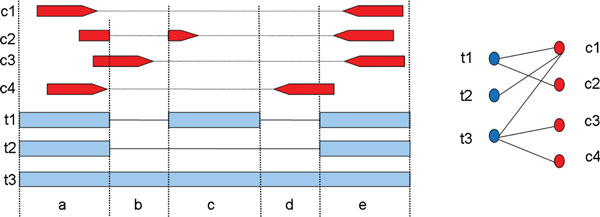
**Constraint graph for four read pairs *c*_1_, *c*_2_, *c*_3_, *c*_4 _and three predicted transcripts *t*_1_, *t*_2_, *t*_3_**.

For clarity, we first describe a bipartite graph model for selecting transcripts and then show how to transform it into an instance of SET_COVER. Reads and read pairs introduce *constraints *on the sets of exons that can be assembled into transcripts, including fragment length constraints as defined earlier and constraints related to the co-inclusion of subexons. We represent each constraint derived from a read, or pair of reads, as the set of intervals that overlap the read(s); multiple reads or read pairs may then produce the same constraint. We consider a bipartite graph (C∪T,ε), where  C is the set of constraints as defined above and  T is the set of candidate transcripts, and we establish an edge (c,t)∈εif transcript *t *satisfies constraint *c*. One example of constraint graph for four read pairs *c*_1_, *c*_2_, *c*_3_, *c*_4 _and three transcripts *t*_1_, *t*_2_, *t*_3 _is shown in Figure 2. Constraints that are not satisfied by any transcript, which are likely to represent artifacts, are removed. The solution to the transcript selection problem can then be formulated as the smallest set of transcripts that collectively satisfy all reachable constraints.

It is easy to see how this can be formulated as an instance of the SET_COVER problem: each candidate transcript *t *corresponds to the set of constraints C⊆C it satisfies. We solve using the following *ln*(|*C*|/*OPT*) greedy approximation algorithm [13]:

Set Cover (C,T)

1. *S *← ∅

2. While  C contains elements not covered by *S*:

(a) Find transcript *t *containing the largest number of constraints not satisfied by transcripts in *S*

(b) Add *t *to *S*

*Incorporating gene structure knowledge into transcript selection*. The above criterion simply minimizes the number of selected transcripts. However, the modular organization of biological sequences into protein domains and regulatory blocks implies that local exon-intron substructures are likely to recur among isoforms. We formulate the *weighted *SET_COVER problem for transcript selection by assigning a weight (cost) to each transcript and then solving with a similar greedy approximation algorithm [13]. We implemented a simple weight function that first determines the number of pairs of consecutive introns in the candidate transcript that can be found in cDNA sequences [14], then assigns a weight to the transcript proportional to its fraction of intron pairs not covered by the measure. We are exploring more sophisticated weight functions that combine several evidence-based criteria, similar to those we used in [9], for future implementations. We call the weighted version of the program CLASS, and the unweighted version CLASS0.

## Comparative evaluation

### Evaluation against a gold reference

We evaluated our methods and three other leading transcript assemblers (Cufflinks [5], IsoCEM [7] and Scripture [6]) on simulated data, which allows us to precisely assess their accuracy relative to a gold reference. We used default values for IsoCEM (version 0.9) and Scripture (version beta-2), and option *'-F 0.01' *for Cufflinks (version 2.0.2), since it was significantly more sensitive than the default version in previous testing [15]. Using the program FluxSimulator [16], we generated 140 million 75 bp paired-end reads and, separately, 140 million 75 bp single-end reads, using the ENSEMBL 61 annotation as model (120,221 reference transcripts). FluxSimulator first assigns an expression level (possibly 0) to each transcript in the annotation, and then simulates all steps in the library preparation process in a typical RNA-seq experiment. Fragments generated are then sequenced *in silico *from one or both ends to generate single-end and paired-end reads, respectively. No sequencing errors were introduced, but reads were then mapped to the human genome hg19 using Tophat [17], a spliced read mapper, which can introduce mismapping artifacts in the assembly process.

For simplicity, we restricted our analysis to chromosome 12, which left 3,281,440 paired-end reads and 3,400,225 single-end reads. Reads were assembled with each of the four programs. Running times were roughly comparable among programs (paired-end reads: 1m45s IsoCEM, 9m14s Cufflinks, 1m17s Scripture, 2m24s CLASS and 2m10s CLASS0; single-end reads: 1m25s IsoCEM, 9m13s Cufflinks, 1m6s Scripture, 2m32s CLASS, 2m47s CLASS0), and therefore will not be discussed further.

We compared the results of each program against the gold reference at exon and transcript levels, using the following criteria. To assess the accuracy of *exon *reconstruction, we consider a match if: *i*) an internal predicted exon matches an internal exon in the annotation *precisely *at both ends; *ii*) a terminal predicted exon matches an annotation exon at the splice site end, and is included in the reference exon; and *iii*) an exon not bounded by splice sites is included in an annotation exon.

Similarly, a predicted *transcript *is said to match a reference isoform if all of its internal introns appear consecutively in the reference. Because transcripts may be only partially sampled and/or reconstructed, we also calculate an effective *coverage *value for both reference and predicted transcripts, defined as the fraction of the reference transcript's exons in the longest match for the transcript being assessed.

With these definitions, we use the conventional *recall *(sensitivity) and *precision *indicators to assess the accuracy of assemblies:

(1) $$Recall\;=\;\frac{K}{M}$$

(2) $$Precision\;=\;\frac{K^{\prime }}{N}$$

if *K *out of *M *reference transcripts match *K*' out of *N *candidate transcripts, as defined in [7]. Since programs may produce partial isoforms, we use precision and recall curves to plot these values as we vary the effective coverage cutoff, as defined above.

**Figure 3:**
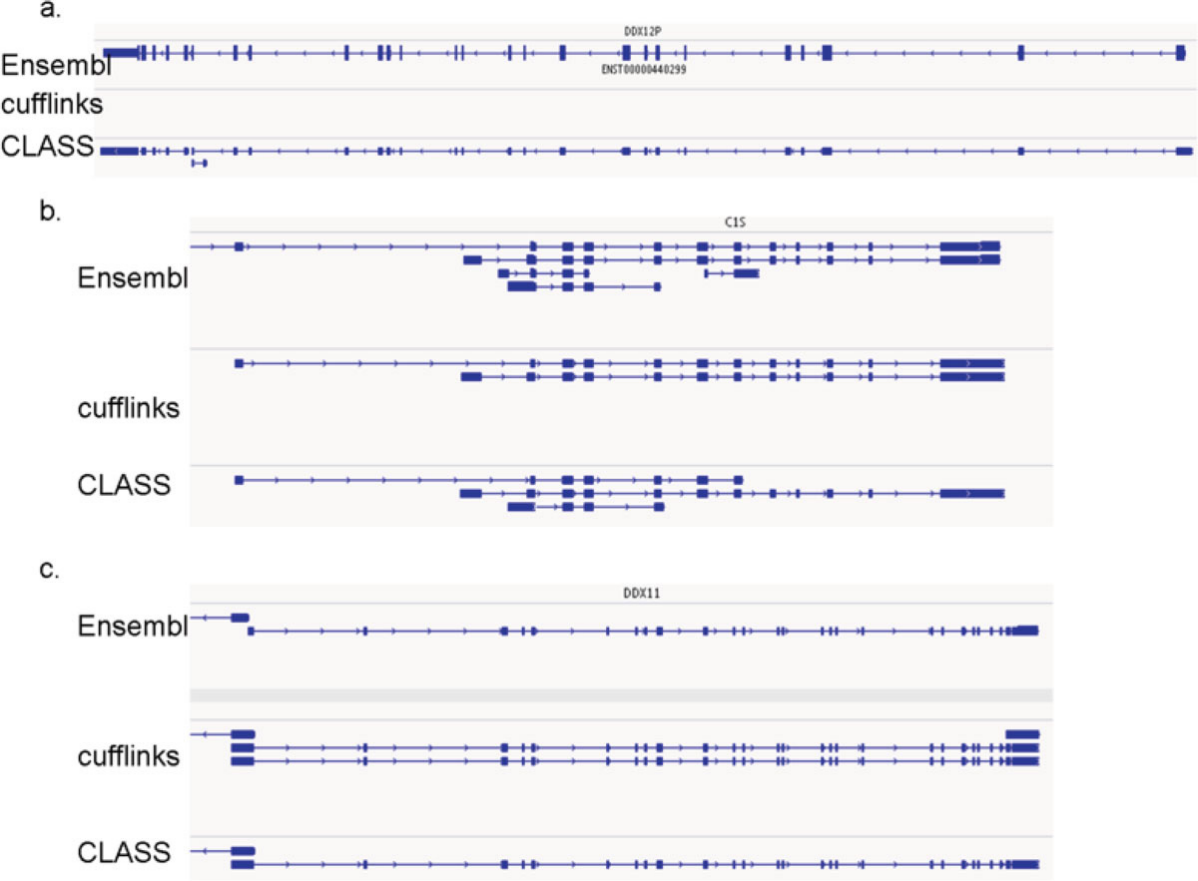
**Examples of CLASS predictions outperforming other programs' on the simulated single-end read data**. (a) Cufflinks fails to predict a transcript at the *DDX12P *gene locus, whereas CLASS predicts the full transcript. (b) CLASS finds more of the splice forms, including alternative 5' and 3' terminal exons, for the *C1S *gene. (c) Cufflinks produces spurious isoforms, including a short single-exon transcript and a 5 bp variation on exon 22, for the *DDX11 *gene. The reference ENSEMBL transcripts sampled by the reads are shown in the top panels.

**Figure 4:**
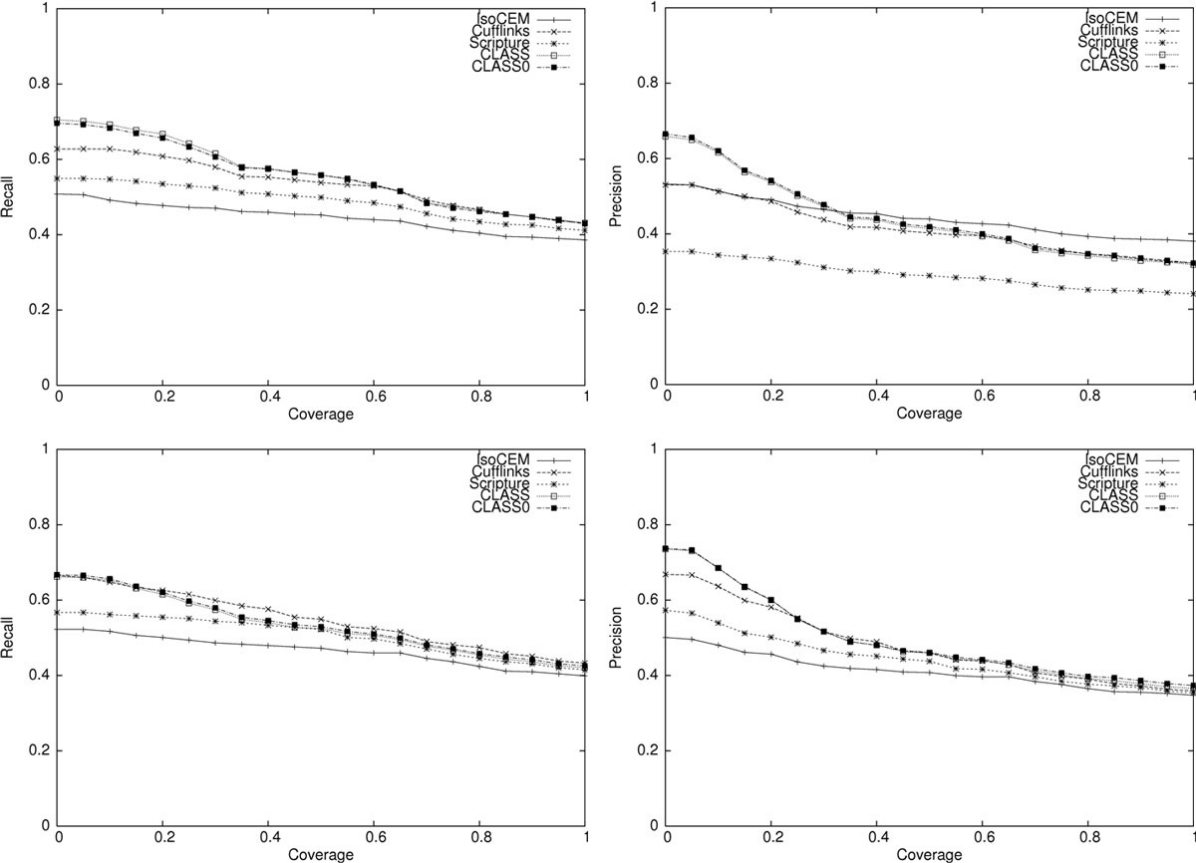
**Performance curves of four programs on simulated reads: paired-end (top) and single end (bottom)**.

The results of the comparison for the two simulated data sets are shown in Table 1. CLASS and CLASS0, implementing the weighted and the unweighted version of SET_COVER, clearly outperform the other programs tested in both sensitivity (0.2-20% higher than its competitors) and precision (7-31% higher) at transcript level, and similarly at exon level. (Several examples illustrating scenarios where CLASS outperforms other methods are presented in Figure 3.) Moreover, they are comparable or better for any any coverage cutoff (Figure 4). We note that CLASS0 edges out CLASS in overall performance, however this is an artifact of producing a slightly smaller number of transcripts, which increased its precision despite having fewer transcripts matched to the reference.

**Table 1:** Accuracy evaluation on simulated data.

Set	Exons					Transcripts				
	**Total**	**Match_ref**	**Match_pred**	**R**	**P**	**Total**	**Match_ref**	**Match_pred**	**R**	**P**

ENSEMBL	4401	-	-	-	-	559	-	-	-	-

Paired-end reads										

IsoCEM	3070	2231	2230	0.507	0.726	562	284	299	0.508	0.532
Cufflinks	3680	2515	2522	0.571	0.685	738	351	391	0.628	0.530
Scripture	3347	2385	2394	0.542	0.715	954	307	337	0.549	0.353
CLASS	3639	2685	2707	0.610	0.744	748	394	493	0.705	0.659
CLASS0	3639	2685	2707	0.610	0.744	735	389	489	0.696	0.665

Single-end reads										

IsoCEM	3114	2242	2247	0.509	0.722	633	292	317	0.522	0.501
Cufflinks	3423	2539	2593	0.577	0.746	666	372	445	0.665	0.668
Scripture	3156	2374	2401	0.539	0.761	658	317	377	0.567	0.573
CLASS	3466	2668	2683	0.606	0.774	635	370	467	0.662	0.735
CLASS0	3466	2668	2683	0.606	0.774	629	373	464	0.667	0.738

Abbreviations: *Match_ref *= reference transcripts (exons) that match predictions; *Match_pred *= predicted transcripts (exons) that match the reference; *R *= recall; *P *= precision.

While the analysis above captures the performance of CLASS in predicting exons, we further sought to separately assess the contribution of its transcript selection process to the overall program performance. We applied the CLASS transcript selection algorithm to the exon sets produced by each of the other three programs (Table 2 and comparison with Table 1). Sensitivity and precision values varied slightly, within two percentage points, for Cufflinks and IsoCEM, suggesting that exon prediction is primarily responsible for CLASS performing better. For Scripture, however, precision was significantly improved for a very slight loss in sensitivity, indicating that the parsimonius approach taken by CLASS is better suited than Scripture's combinatorial approach for these data sets.

**Table 2:** Performance of CLASS with alternate exon data.

Set	Transcripts	Match_ref	Match_pred	Recall	Precision
Paired-end reads					

CLASS_IsoCEM	541	279	293	0.499	0.542
CLASS_Cufflinks	744	343	389	0.614	0.523
CLASS_Scripture	622	297	325	0.531	0.523

Single-end reads					

CLASS_IsoCEM	614	286	311	0.512	0.507
CLASS_Cufflinks	666	366	436	0.655	0.655
CLASS_Scripture	568	303	361	0.542	0.636

### Evaluation on real data

To assess the practicality of using the program in large RNA-seq applications, we applied CLASS to the 160 million 50 bp paired-end reads from Illumina's Human Body Map adrenal tissue sample. Again, we restricted our analysis to chromosome 12, with 3,280 spliced genes. Because the ENSEMBL annotation is inherently incomplete and may also include genes and isoforms not present in adrenal tissue, it is not possible to determine the programs' true sensitivity and precision. Nevertheless, consistency with the reference annotation, in particular sensitivity, provides a good indication of a program's performance. Because now both the reference and the reconstructed transcripts may be incomplete, we relax the definition of a match to include all pairs of reference and candidate transcripts with compatible intron patterns, using the effective coverage defined above to more finely explore the extent of their agreement. In addition, we allow for a small margin of error at exon boundaries (*V *= 10) to account for potential inaccuracies in the reference annotation.

Both Cufflinks and IsoCEM predicted a very large number of transcripts (Table 3), which dramatically reduced their precision. Cufflinks found the most reference transcripts, however, we correctly hypothesized that many of these were due to single exon assemblies. Because most of single exon assemblies are biological or computational artifacts which are usually filtered out during transcriptome analysis, we removed them from all data sets. This significantly improved IsoCEM, Cufflinks and Scripture's precision, while values for CLASS and CLASS0 were robust. Moreover, both before and after filtering, CLASS and CLASS0 achieve the highest overall accuracy as measured by the F-value (Table 3):

**Table 3:** Performance of four programs on the adrenal data set

	All				Multi-exon only			
**Set**	**Transcripts**	**Recall**	**Precision**	**F-value**	**Transcripts**	**Recall**	**Precision**	**F-value**

ENSEMBL	3280	-	-	-	3280	-	-	-
IsoCEM	21339	0.696	0.137	0.229	1951	0.535	0.765	0.630
Cufflinks	13073	0.798	0.318	0.455	3316	0.637	0.729	0.680
Scripture	11553	0.638	0.472	0.543	7573	0.539	0.561	0.550
CLASS	7394	0.745	0.800	0.772	5117	0.704	0.795	0.747
CLASS0	7062	0.744	0.786	0.764	4785	0.703	0.775	0.737

F=2*Recall*Precision/(Recall+Precision)

**Figure 5:**
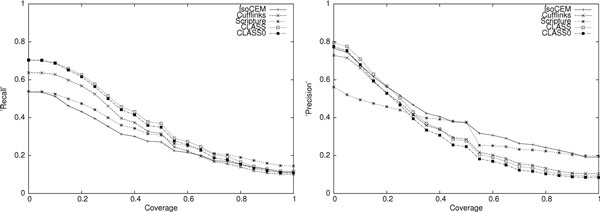
**Performance curves of four programs on the adrenal data set, on multi-exon transcripts only**.

To draw a more detailed picture of programs' accuracy, we plotted the recall and precision when varying the coverage cutoff. As Figure 5 shows, CLASS and CLASS0 consistently achieve the highest sensitivity, by finding more of the reference transcripts. In contrast, IsoCEM is more precise than any of its competitors at almost any coverage cutoff, but its sensitivity, or the actual number of reference transcripts found, is small (e.g., 1,756 compared to 2,310 for CLASS).

## Conclusions

We present a novel algorithm and computer program for assembling transcripts from RNA-seq data, combining a linear program to infer exons with a transcript selection scheme that determines the final set of transcripts based on contiguity constraints derived from spliced and paired reads and on gene structure knowledge available from cDNA sequence databases. CLASS outperformed Cufflinks, IsoCEM and Scripture, three of the leading transcript assembly programs, in overall accuracy on both simulated and real data and, unlike other programs that report significant amounts of 'noise', it provided a robust and easy to interpret set of transcripts. Further improvements in the algorithm and implementation, including more sophisticated weight functions, will increase the program's accuracy and speed, and implicitly its usefulness for annotation.

## Competing interests

The authors declare that they have no competing interests.

## Authors' contributions

LF and LS designed the algorithm and LS implemented it in software. Both authors contributed to analyses and prepared the manuscript.

## Declarations

Funding for publication of this article was provided by NSF (ABI award 1159078).

This article has been published as part of *BMC Bioinformatics *Volume 14 Supplement 5, 2013: Proceedings of the Third Annual RECOMB Satellite Workshop on Massively Parallel Sequencing (RECOMB-seq 2013). The full contents of the supplement are available online at http://www.biomedcentral.com/bmcbioinformatics/supplements/14/S5.
